# Understanding bracing outcomes in adolescents with idiopathic scoliosis: a mixed-methods approach

**DOI:** 10.3389/fresc.2025.1625736

**Published:** 2025-07-23

**Authors:** Hasan İşçi, Sena Özdemir Görgü

**Affiliations:** ^1^ Department of Orthotics and Prosthesis, Institute of Health Sciences, Istanbul Medipol University, Istanbul, Turkiye; ^2^ Department of Orthotics and Prosthesis, Faculty of Health Sciences, Istanbul Medipol University, Istanbul, Turkiye

**Keywords:** adolescent idiopathic scoliosis, spinal brace, brace satisfaction, quality of life, user experience, parental perspective

## Abstract

**Background:**

Brace treatment is the routinely applied conservative treatment method for Adolescent Idiopathic Scoliosis (AIS) and can yield effective results when correctly administered. Brace treatment, often initiated during adolescence, can cause individuals to face various challenges in their daily lives. This study determined the effect of spinal brace treatment on the quality of life (QoL), views, and the problems encountered regarding bracing by individuals with AIS and their parents.

**Methods:**

This study included 44 participants aged 10–17 years using spinal braces for at least six months. Participants' QoL was assessed using the Brace Questionnaire (BrQ), a numerical evaluation scale for brace satisfaction (BS), and the extracted user and parental opinions to identify the problems affecting brace use. Individual interviews were conducted with selected groups of ten participants to examine thoroughly the factors influencing spinal brace use.

**Results:**

Pearson correlation analysis revealed positive correlation between BS and total BrQ scores and sub-parameters the regression analysis to determine the cause-and-effect relationship between BS and total BrQ scores with sub-parameters was significant (F = 7.648, *p* = 0.008; *F* = 2.935, *p* = 0.015, respectively). Regarding brace satisfaction and influencing factors, most participants reported breathing difficulties, dissatisfaction with the weight, color, and design of the brace, feeling chest pain, and experiencing balance problems on surveys and individual interviews.

**Conclusions:**

This study established a correlation between BS and QoL and identified the design, weight, and material-related issues of the orthosis as significant factors influencing brace satisfaction in individuals using spinal braces.

## 1 Introduction

Idiopathic scoliosis is the most common spinal deformity in adolescents, with a prevalence of 2%–4% in the general population (1). The treatment of Adolescent Idiopathic Scoliosis (AIS) is determined based on the severity of the spinal curvature and may include observation, exercise, brace therapy, or surgical intervention (2, 3). The goals of scoliosis treatment are to correct spinal deformities early, prevent curvature progression, alleviate pain, and enhance patients' quality of life (QoL) (3).

Brace treatment is recommended for individuals with a Cobb angle between 20 and 45 degrees who have not yet reached skeletal maturity. Brace treatment is the routinely applied conservative treatment method for AIS and can yield effective results when correctly administered (2, 4). The multicenter BrAIST trial conducted by Weinstein et al. (2013) demonstrated that bracing significantly reduces the risk of curve progression to the surgical threshold (Cobb angle of ≥50 degrees) in high-risk adolescents with idiopathic scoliosis (5).

The application of a variety of brace concepts, distinguished by the prescribed wearing time and rigidity of the brace, has been described in the literature. These concepts include full-time, part-time, and night-time braces, as well as rigid and soft braces (6). Despite using various brace designs in AIS treatment, the fundamental objective is to restore normal spinal alignment and correct the aesthetic appearance through external forces (4, 6, 7). A review of the literature reveals a number of studies that attest to the efficacy of braces in preventing the progression of curvature and even in reducing their severity (8). A recent systematic review has demonstrated that bracing may be an effective intervention for patients with AIS curves ≥40° who are unwilling to undergo posterior spinal fusion (9).

Brace treatment, often initiated during adolescence, can cause individuals to face various challenges in their daily lives (3). The success of the brace depends on the specific design tailored to the treated curvature, the follow-up process, the patient's compatibility with and adherence to the brace, and the duration of wear. In the literature, QoL assessments have been based on the perspectives of individuals using brace, and reliability of these studies relies solely on the responses provided by users. There are limited studies in the literature that explore the opinions of both brace users and their parents. We also aimed to understand the views of individuals with AIS and their parents regarding brace use, including the difficulties they experienced, the negative impacts they perceived, and the complaints they expressed throughout the treatment process.

## 2 Materials and methods

This study was a prospective explanatory sequential mixed-methods investigation. Quantitative data were collected and analyzed first, followed by qualitative data obtained through in-depth individual interviews. This design was adopted to gain a deeper understanding of the quantitative findings concerning the impact of brace treatment on the QoL individuals with AIS. The Istanbul Medipol University Ethics Committee approved the study (decision number 946, E-10840098-772.02-66608, dated 30.12.2020), which was conducted between January 2021 and April 2021. The research was carried out in accordance with the Declaration of Helsinki.

### 2.1 Participants

**Figure 1:**
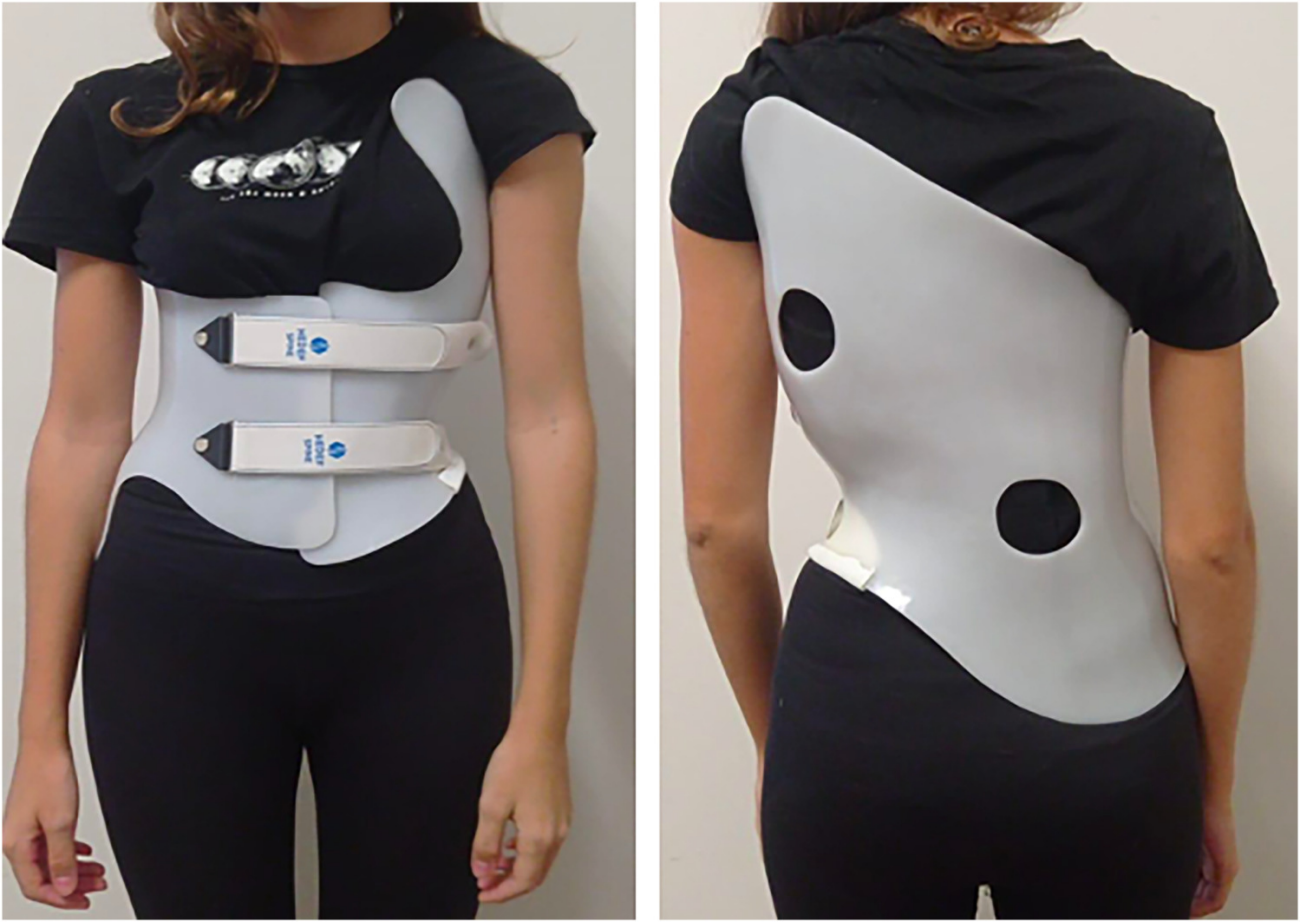
Rigo-Chêneau brace produced using CAD-CAM technologies.

All spinal orthoses used in the study were initially prescribed by orthopedic specialists and subsequently fitted by a certified orthotist at a single specialized scoliosis center located in Istanbul, Turkey. In line with the medical indication, all participants received a Rigo-Chêneau brace, which is a three-dimensional thoracolumbosacral orthosis (TLSO) specifically designed to provide asymmetric pressure and expansion areas tailored to individual curve patterns. Each orthosis was custom-designed using surface topography and clinical measurements obtained while patients were standing in an uncorrected posture, with the aid of a Structure Sensor. The digital data were processed and modified using Meshmixer software by a certified clinician at the Istanbul Hedef Spine Clinic. The final braces were produced using computer-aided design (CAD) and computer-aided manufacturing (CAM) technologies (10). The entire orthotic design, production, and fitting process was performed at a single, specialized spinal brace fitting center (Istanbul Hedef Spine Clinic) to ensure consistency, standardization, and patient-specific customization. A Rigo-Chêneau brace fabricated using CAD-CAM technologies is illustrated in Figure 1.

**Figure 2:**
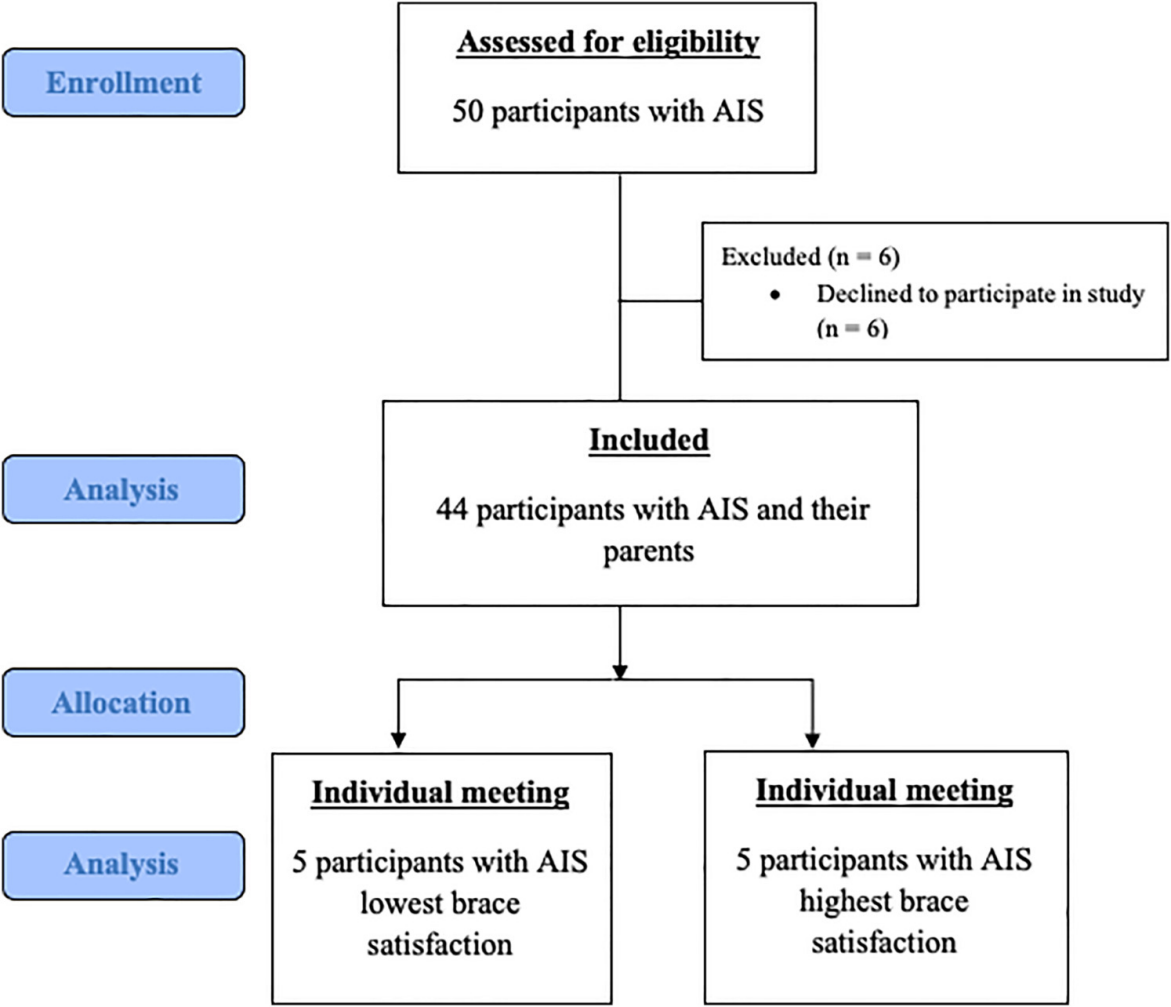
Participants' selection flowchart.

The inclusion criteria for this study were as follows: confirmed AIS diagnosis, brace treatment for at least 6 months, and living with family. Participants with neurological or mental disorders, a history of spine surgery, and parents with mental or vision disorders were excluded from the study. In total, 44 individuals with AIS and their parents who met the inclusion criteria were included in this study. Signed informed consent was obtained from all the participants with AIS and their parents. A flowchart illustrating the selection of participants is shown in Figure 2.

### 2.2 Study interventions

General information on the individuals diagnosed with AIS using braces included in the study was collected through a demographic information form. The duration of brace use (in months) and the average daily wear time were obtained through self-reports provided by participants and their parents during interviews and questionnaire completion. Similarly, Cobb angle measurements were clinically performed by orthopedic specialists during routine follow-ups; in this study, these values were reported by the parents based on their most recent medical evaluations. The Brace Questionnaire (BrQ) was used to assess their QoL (11), and a numerical rating scale was employed to evaluate brace satisfaction (BS). An evaluation form was used in this study to obtain the opinions of individuals with AIS using braces and their parents regarding the brace. In the qualitative part of the study, semi-structured individual interviews were conducted to inquire about the participants' opinions on the brace.

The BS was utilized to assess the orthotic satisfaction of individuals using braces. Participants were requested to rate their overall satisfaction with their braces in terms of ease of donning and doffing, comfort, aesthetic appearance, weight, skin irritation and damage to clothing on a scale of 0–10 (0 = not satisfied at all; 10 = very satisfied), with higher scores indicating greater satisfaction with the brace (12).

The BrQ was employed to assess the QoL associated with brace use in individuals with AIS. It comprised 34 questions under 8 subheadings: general health perception, physical function, emotional function, pain, school life, social life, vital signs, and self-esteem. Responses to questions are given on a five-point Likert scale (“Never, Rarely, Sometimes, Often, Always”). The total score ranged from 20–100 points, with higher scores indicating higher QoL (11, 13).

Participants' opinions on braces and the difficulties they experienced using braces in their daily lives were queried using the Participant Evaluation Form (PEF) created by the researchers. The PEF is a 13-question form developed by researchers based on their clinical experiences, expert clinician opinions, and a literature review (see Supplementary Appendix 1). The parental evaluation form (PAEF) was used to inquire about difficulties participants experience with braces in their daily lives, observations of their parents, their opinions on braces, and whether individuals adhere to daily brace usage protocols. The PAEF, consisting of 10 questions, was created by researchers based on their clinical experience, expert clinician opinions, and a literature review (Supplementary Appendix 2).

The qualitative part of our study was conducted through semi-structured individual interviews to examine the factors influencing brace satisfaction in individuals with AIS undergoing brace treatment. The questions posed in the interviews were constructed based on the clinical experiences of the researchers, expert opinions, and a comprehensive literature review (Supplementary Appendix 3). In order to ascertain the suitability of the draft questions, the opinion of an expert in the field was sought. Individual interviews, an average of 15 min, were conducted under parental supervision. Individual interviews involved obtaining consent from individuals with AIS and their families; assessments were completed by voice recording and evaluating the interviews.

### 2.3 Statistical analysis

To determine the required sample size, calculations were performed using G*Power 3.1 software. A comparable study by Vasiliadis and Grivas (2008) reported moderate positive correlations between corset satisfaction and quality of life domains, with correlation coefficients ranging from *r* = 0.31 to *r* = 0.512. Based on these findings, a power analysis was conducted (14). A one-tailed test was utilized, with the alternative hypothesis correlation (ρH1) set at 0.512. The significance level (*α*) was fixed at 0.05, and the desired statistical power (1-β) was set at 0.95. The null hypothesis correlation (ρ H0) was assumed to be 0. The analysis yielded lower and upper critical *r* values of 0.278. It was determined that a minimum sample size of 36 participants was required to achieve the desired 95% statistical power. To account for a potential dropout rate of 20%, the study was conducted with a total of 44 participants. Frequencies, percentages, mean, and standard deviation in descriptive statistical methods were used to summarize data using SSPS for Windows 22.0. Pearson's correlation analysis was conducted to assess the relationships between continuous variables. Data are presented as arithmetic mean and standard deviation (Mean ± SD). Statistical significance was set at *p* < 0.05. tests.

To prevent bias in the qualitative phase, semi-structured interviews were conducted with individuals exhibiting varying levels of satisfaction. Specifically, 10 participants with AIS were purposively selected based on their brace satisfaction (BS) scores; five with the lowest scores (Group A) and five with the highest (Group B) (15). All recorded interviews were transcribed verbatim for analysis. Descriptive analysis, a qualitative method focused on summarizing data according to pre-established themes, was employed to interpret the findings (16). To assess the reliability of the coding process, the inter-coder reliability formula by Miles and Huberman (1994) was applied: reliability = agreements/(agreements + disagreements). The analysis yielded an agreement rate of 89%, which exceeds the commonly accepted threshold of 80%, indicating that the coding process in this study was methodologically sound (17).

## 3 Results

The demographic information of the participants, along with the mean values of the BS, BrQ (total), and the subparameters of the participants, is displayed in Table 1.

**Table 1:** Baseline characteristics, brace satisfaction, and quality of life scores of study participants.

Variable	*N*	%
Gender	44	
Male (*n* = 3)		6.8%
Female (*n* = 41)		93.2%
Type of spinal brace		
Rigid	44	100%
Major curve		
Thoracal (*n* = 30)		68.2%
Thoracolumbar (*n* = 6)		13.6%
Lumbar (*n* = 8)		18.2%
Variable	Mea*n* ± SD	Min–Max
Age (year)	13.71 ± 1.35	11–17
Height (cm)	162.82 ± 7.19	145–186
Weight (kg)	47.93 ± 7.91	32–67
BMI (kg/m^2^)	18.04 ± 2.47	13.76–23.18
Brace usage time (months)	18.27 ± 17.77	6–84
Daily brace usage time (hours)	20.42 ± 2.7	10–24
Current cobb angle	30.86 ± 9.38	10–50
BS	6.91 ± 2.36	0–10
BrQ		
Total score	76.71 ± 12.29	42–95
General health perception	3.07 ± 1.01	1–5
Physical functioning	3.86 ± 0.62	2–5
Emotional functioning	3.57 ± 0.82	1.2–5
Self-esteem and esthetics	3.52 ± 0.97	1–5
Vitality	3.47 ± 0.96	1–5
School activity	4.08 ± 0.89	2–5
Bodily pain	3.38 ± 0.53	1.8–4.1
Social life	3.97 ± 0.82	1.7–5

N, Total number of participants; %, Percentage; *n*, Number of participants; SD, standard deviation; Min, minimum; Max, maximum; BMI: body mass index; BS, brace satisfaction scale; BrQ, brace questionnaire.

Pearson's correlation analysis between BS and BrQ revealed a positive correlation between the BrQ total score and the sub-parameters of physical function, emotional function, self-esteem, vitality, bodily pain, and social life with BS (*p* = 0.008, *p* = 0.038, *p* = 0.000, *p* = 0.007, *p* = 0.009, *p* = 0.017, *p* = 0.045, respectively). The correlation analysis results for BS and BrQ are presented in Table 2.

**Table 2:** Results of the correlation analysis between brace satisfaction and quality of life.

BS	BrQ								
	Total	General health perception	Physical functioning	Emotional functioning	Self-esteem	Vitality	School activity	Bodily pain	Social life
r	0.389**	0.108	0.310*	0.512**	0.400**	0.383**	0.009	0.355*	0.300*
p	0.008	0.482	0.038	0.000	0.007	0.009	0.953	0.017	0.045

BrQ, brace questionnaire; BS, brace satisfaction scale.

* <0.05.

** <0.01.

The results of the first 12 questions of the PEF are presented in Table 3. In response to the 13th question, which addressed participants' exercise programs, 84% (*n* = 37) of participants mentioned the Schroth method, 5% (*n* = 2) mentioned Pilates, and 11% (*n* = 5) reported receiving only brace treatment. The results of the four questions of the PAEF, evaluated using a 5-point Likert scale, are also presented in Table 3. Regarding the relationship of the interviewees to individuals with AIS, 80% (*n* = 35) were mothers, 16% (*n* = 7) were fathers, and 4% (*n* = 2) were others. When asked about the presence of scoliosis or spinal deformities in other siblings, 82% (*n* = 36) reported “no,” while 18% (*n* = 8) reported “yes.” In the 10th question, when parents were asked about the reasons for their children's unwillingness to use a brace, 18% (*n* = 8) mentioned difficulty in breathing, 27% (*n* = 12) reported problems in social activities, 7% (*n* = 3) indicated sleep problems, 16% (*n* = 7) reported material discomfort, 9% (*n* = 4) felt embarrassed while using the brace, 2% (*n* = 1) stated difficulty attending to toilet needs, and 27% (*n* = 12) reported excessive sweating. Additionally, 24% (*n* = 11) indicated that their children had no issues using the brace.

**Table 3:** The results of the participant and parental evaluation forms.

Participants' evaluation results					
	Never	Almost never	Sometimes	Often	Always
	*n* (%)	*n* (%)	*n* (%)	*n* (%)	*n* (%)
I can put on my brace by myself	2 (4.5%)	2 (4.5%)	5 (11.4%)	2 (4.5%)	33 (75%)
I wear my brace for the planned duration	0	0	5 (11.4%)	13 (29.5%)	26 (59.1%)
I have difficulty breathing with my brace on	0	5 (11.4%)	13 (29.5%)	15 (34.1%)	11 (25%)
I have issues sleeping with my brace on	2 (4.5%)	2 (4.5%)	11 (25%)	12 (27.3%)	17 (38.6%)
I have difficulty eating with my brace on	4 (9.1%)	9 (20.5%)	12 (27.3%)	7 (15.9%)	12 (27.3%)
I can comfortably use the toilet with my brace on	9 (20.5%)	3 (6.8%)	6 (13.6%)	8 (18.2%)	18 (40.9%)
The materials attached inside my brace cause itching/redness	5 (11.4%)	8 (18.2%)	11 (25%)	13 (29.5%)	7 (15.9%)
I am not satisfied with the color of my brace	6 (13.6%)	1 (2.3%)	3 (6.8%)	2 (4.5%)	32 (72.7%)
My brace feels heavy to me	0	2 (4.5%)	10 (22.7%)	10 (22.7%)	22 (50%)
I feel pain in my chest area	1 (2.3%)	0	16 (36.4%)	9 (20.5%)	18 (40.9%)
I have balance problems with my brace on	1 (2.3%)	0	11 (25%)	10 (22.7%)	22 (50%)
I am satisfied with the exercise program	4 (9.1%)	0	4 (9.1%)	8 (18.2%)	28 (63.6%)
Parentals' evaluation results					
Is your child willing to do the exercises?	3 (6.8%)	4 (9.1%)	10 (22.7%)	14 (31.8%)	13 (29.5%)
Does your child comply with the daily brace wearing duration?	0	0	2 (4.5%)	15 (34.1%)	27 (61.4%)
Does your child complain about the brace?	1 (2.3%)	5 (11.4%)	24 (54.5%)	11 (25%)	3 (6.8%)
Is your child willing to wear the brace?	1 (2.3%)	5 (11.4%)	12 (27.3%)	19 (43.2%)	7 (15.9%)

*n*, Number of participants; %, Percentage.

**Figure 3:**
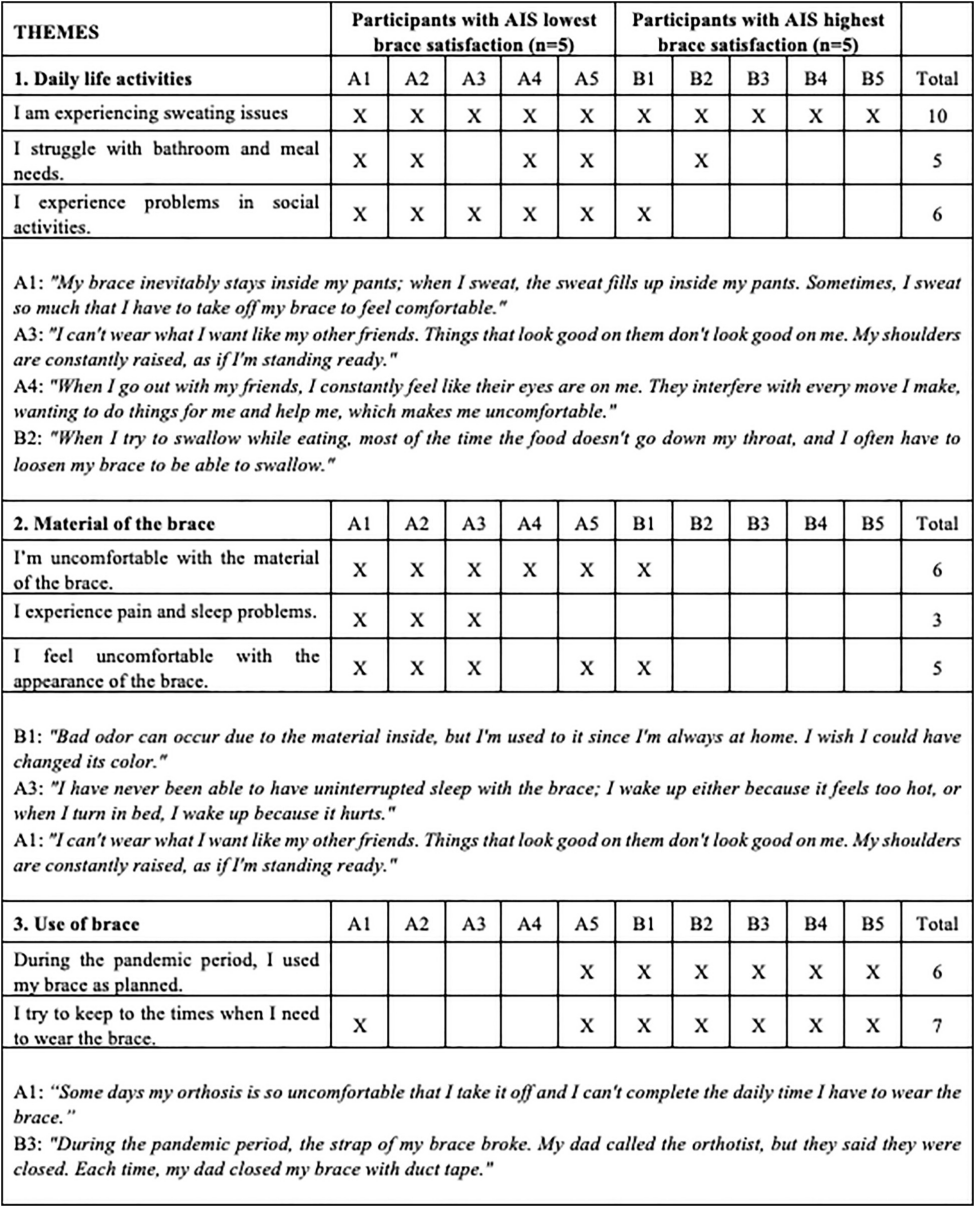
Semi-structured individual interview results [each cross **(X)** indicates that the participant reported experiencing the corresponding issue. The totals reflect the number of participants (out of 10) who confirmed each issue].

In the qualitative part of the study, the responses to the individual interviews conducted using interview forms were analysed under three themes in order to examine the factors affecting brace satisfaction in individuals with AIS receiving brace treatment. These themes are as follows: opinions related to daily life activities involving orthosis use; opinions about the material of the orthosis; and opinions about the use of orthosis. The results of the semi-structured individual interviews are shown in Figure 3.

## 4 Discussion

This study evaluated the impact of orthotic treatment on the QoL, recorded opinions, and problems encountered regarding braces by individuals with AIS using braces for at least 6 months and their parents. Our study results indicated that the QoL of individuals with AIS improved with increased brace satisfaction. Furthermore, brace design, weight, and materials, leading to problems such as sweating, balance issues, and chest pain, were the most significant factors affecting brace satisfaction. In the qualitative part of the study, all the participants reported sweating caused by the closed brace design as the most significant issue. They mentioned having to change clothes constantly because of the excessive sweating caused by the brace, which caused problems. Individuals using braces for 6–7 months reported pain in the chest and axillary region, and those using braces for 8–18 months mentioned sleep problems and difficulty during various daily activities (eating, using toilets, sitting, and standing). They also pointed out that using plastazole for pads causes sweating, odor, and dermal problems. The participants especially complained about the color of the brace, with all participants using white orthoses expressing a preference for darker colors.

Literature supports brace treatment as an effective and conservative method for stopping or reducing the progression of scoliosis in AIS (5). Despite the positive effects of brace treatment, it causes stress in individuals and has a significant negative impact on their QoL (18, 19). Design and rigidity (soft, semirigid, and rigid) of braces in individuals with AIS have different effects on QoL (2, 6). Our study participants used rigid braces, and their QoL and brace satisfaction were above average, with a positive relationship identified between brace satisfaction and QoL (20). Additionally, brace satisfaction was revealed to have a determining effect on QoL, physical function, emotional function, self-esteem, vital signs, school life, pain, and social life. Those with AIS who experience difficulties or challenges while wearing a brace have a detrimental effect on their quality of life. Thus, it can be concluded that individuals with AIS experience better QoL with increasing duration of brace use (in months), provided that brace satisfaction is maintained.

Brace treatment has been shown in the literature to be directly correlated with treatment effectiveness when the brace is worn consistently for the recommended duration (21). Furthermore, recent studies have highlighted that in-brace correction is a key predictor of treatment success, as greater initial correction may enable reduced wear time while still achieving comparable clinical outcomes and improved quality of life (22, 23). In our study, daily brace wear time was reported by participants and their parents through self-report. It is important to note that previous research has demonstrated that self-reported wear time often overestimates actual brace usage when compared to objective monitoring tools such as temperature or force sensors (24). Despite this, participants in our study reported wearing the brace for an average of 20 h per day over a period of 18 months, indicating high compliance. This was associated with observed improvements in Cobb angles. Our findings further suggest that long-term brace use did not negatively impact quality of life (18), supporting the view that consistent compliance, especially when combined with effective in-brace correction, may help reduce potential negative effects on QoL. Moreover, although our study did not directly investigate psychological coping predictors, recent evidence suggests that variables such as age, gender, and prescribed brace duration may influence individual adaptation to bracing (25). Future research may benefit from exploring these characteristics in relation to brace satisfaction and long-term QoL outcomes.

The brace design, primarily focusing on spinal deformity correction, can lead to physical and aesthetic discomfort in individuals using braces (16, 26). In our study, most participants reported being able to put on their braces independently and use them throughout the planned duration. Their primary complaints related to braces included difficulty breathing, sleep problems, dissatisfaction with brace color, perceiving the brace as heavy, chest pain, and balance problems. Our study results indicate that the participants had physical complaints related to the use of rigid braces, aligning with the literature (27, 28). Aesthetic concerns about the color and/or appearance of braces may stem from the fact that participants used plain white braces, and our country does not commonly apply patterns to braces. In individuals with AIS, providing braces in the desired color/pattern and actively involving them in the brace design may enhance brace compliance, consequently improving treatment effectiveness. In addition to color and pattern preferences, considering the visual design aesthetics of the brace (such as transparency, texture, and shape) may further support user satisfaction and acceptance.

Adolescents with spinal deformities encounter significant obstacles throughout the treatment process, which can ultimately impact the outcomes and the efficacy of brace intervention (29). Active participation of patients and their families in the treatment process is crucial in conservative treatments (30, 31). In our study, the parents of individuals with AIS receiving brace treatment were questioned about their adherence to daily brace use protocols and the difficulties they and their children experienced with bracing in daily life. Parents reported the following as factors affecting brace use; difficulty breathing, problems in social activities, sleep problems, discomfort with the material, feeling ashamed of using brace, difficulty in toilet needs, and sweating. However, 14% of the participants reported no problems related to the brace. Their interview results reveal consistent and common complaints expressed by individuals with AIS and their parents.

All participants lived in Istanbul, Turkey. The city has a humid subtropical climate, characterized by hot and humid summers and mild, rainy winters. In individual interviews, most participants reported excessive sweating and physical discomfort while wearing the brace. While these complaints may be partially attributed to the local climate, we do not consider it to be the sole contributing factor. In addition to climatic influences, the material properties of the braces themselves may also contribute to discomfort, particularly in terms of heat retention and limited breathability. In our study, the Rigo-Chêneau braces were custom-fabricated using 4 mm thick polyethylene for the external shell and 3–4 mm thick plastazote for the internal padding. This material combination is widely used in scoliosis bracing due to its favorable balance between structural support and user comfort. However, to the best of our knowledge, there is limited research specifically addressing the role of materials used in orthotic brace construction in relation to user comfort. Scoliotic braces are composed primarily of thermoplastic materials. Increased sweating is inevitable because of the material thickness and the closed design of the braces. While soft braces are often criticized for being less biomechanically effective (32), we believe that integrating lightweight, breathable, and user-friendly materials into rigid orthosis design without compromising corrective forces could help alleviate user discomfort. For example, plastazole pads commonly used for derotation may cause skin irritation due to their airtight and abrasive nature. Alternative materials and thoughtful design concepts, such as asymmetrical shell structures that eliminate the need for pads, may offer both biomechanical and comfort advantages.

Exercise programs, particularly three-dimensional scoliosis exercises applied with brace therapy, positively affect QoL and Cobb angle (33–36). In our study, participants self-reported their exercise engagement, with 84% (*n* = 37) performing three-dimensional scoliosis exercises and 5% (*n* = 2) reporting Pilates practice, while 11% (*n* = 5) received only brace therapy. These data were collected descriptively and not intended to evaluate the efficacy of individual exercise modalities. Nevertheless, they suggest that exercise, when combined with bracing, may play a role in improving QoL. These findings also reflect the variability of treatment experiences and highlight the importance of a multidisciplinary approach in managing AIS.

Our study indicates that brace therapy positively affects the QoL of individuals with AIS, and brace satisfaction is a significant factor in treatment outcomes. Similar to the suggestion of Joarder et al. of qualitative research yielding more meaningful results with limited quantity, especially in individuals with AIS (37). Similarly, our qualitative research yielded more data, with individuals expressing their problems more effectively. In this context, we believe our study to serve as a guiding resource for clinicians and researchers to focus on the effectiveness of brace treatment and the experiences of patients with AIS.

It is also important to acknowledge that this study was conducted during the COVID-19 pandemic, specifically between January and April 2021, when schools in Turkey were largely operating in a hybrid format and various social restrictions were in place. These circumstances may have had both positive and negative impacts on brace wear time and compliance. For some participants, spending more time at home may have facilitated more consistent brace use, while for others, the lack of daily routine and increased emotional stress may have negatively affected adherence. In addition, Asad et al. identified social criticism as a key stressor in AIS brace treatment (28). In our study, participants reported relatively high satisfaction with brace use in the school context, which may be partly explained by reduced exposure to societal and peer judgment during the period of hybrid education. These contextual factors should be considered when interpreting the findings.

Our study had several limitations, including the inability to compare different brace designs or the effects of short- vs. long-term brace use. Additionally, all assessments were based on self-reported data, which may be subject to recall or reporting bias. The study was also conducted during the COVID-19 pandemic (January–April 2021), when hybrid schooling and social restrictions in Turkey may have influenced participants' routines and brace compliance. These factors should be considered when interpreting the findings.

## 5 Conclusions

In our study, we observed a decrease in complaints as the duration of brace use increased. In particular, the brace design significantly affects orthosis satisfaction. Individual interviews with the participants suggested that increased satisfaction could be achieved by improving the physical characteristics of the brace. Therefore, the development of better braces and treatment strategies has the potential to enhance patients' QoL and treatment effectiveness. Continuous development in this area underscores the importance of prioritizing user feedback and observations from families. We believe the determining factors influencing brace use may positively affect brace compliance, treatment effectiveness, and QoL.

## Data Availability

The datasets generated and/or analyzed during the current study are not publicly available due to privacy and ethical restrictions but are available from the corresponding author upon reasonable request. Audio recordings and qualitative data from individual interviews were obtained with informed consent from individuals with AIS and their families, and are also available upon reasonable request. Requests should be directed to Sena Özdemir Görgü at senaozdemir@medipol.edu.tr.
