# Supplementary material for: Understanding bracing outcomes in adolescents with idiopathic scoliosis: a mixed-methods approach
Source: Front Rehabil Sci. 2025 Jul 23;6:1625736. doi: 10.3389/fresc.2025.1625736 (PMC12325295; doi:10.3389/fresc.2025.1625736)
Supplement: Supplementary file 1 [file Datasheet1.docx]

**PARTICIPANT EVALUATION FORM (PEF)**

|  | Never Almost never Sometimes Often Always |
| --- | --- |
| I can put on my brace by myself | 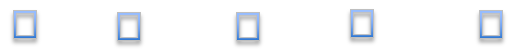 |
| I wear my brace for the planned duration | 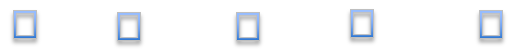 |
| I have difficulty breathing with my brace on | 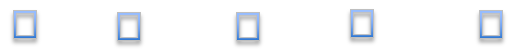 |
| I have issues sleeping with my brace on | 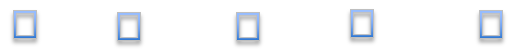 |
| I have difficulty eating with my brace on | 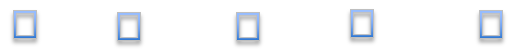 |
| I can comfortably use the toilet with my brace on | 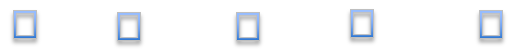 |
| The materials attached inside my brace cause itching/redness | 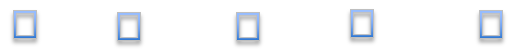 |
| I am not satisfied with the color of my brace | 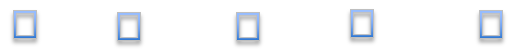 |
| My brace feels heavy to me | 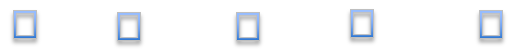 |
| I feel pain in my chest area | 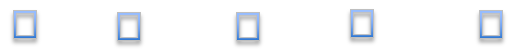 |
| I have balance problems with my brace on | 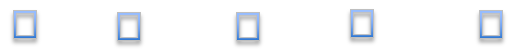 |
| I am satisfied with the exercise program | 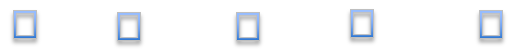 |
| Please write the name of the exercise program you have received for scoliosis treatment. |  |
