# Supplementary material for: Understanding bracing outcomes in adolescents with idiopathic scoliosis: a mixed-methods approach
Source: Front Rehabil Sci. 2025 Jul 23;6:1625736. doi: 10.3389/fresc.2025.1625736 (PMC12325295; doi:10.3389/fresc.2025.1625736)
Supplement: Supplementary file 2 [file Datasheet2.docx]

**PARENTAL EVALUATION FORM (PAEF)**

| Please specify your relationship to the child | Mother Father Other  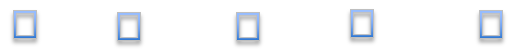 |
| --- | --- |
| What is your highest level of education? | **Elementary ScMiddle Sch. High Sch. B.Sc. M.Sc or PhD**  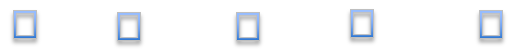 |
| Do any of your other siblings have scoliosis or another spine-related issue? | **Yes No**  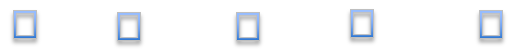 |
| At what age was scoliosis noticed? | **5-9 Age 10-12 Age 13-15 Age Other**  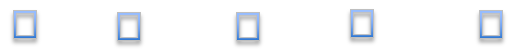 |
| How many months have you been using the spinal brace? | **6-12 13-18 19-24 36-48 60-84**  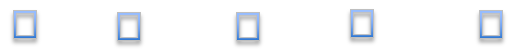 |
|  | **Never Almost never Sometimes Often Always** |
| Is your child willing to do the exercises? | 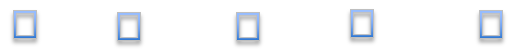 |
| Does your child comply with the daily brace wearing duration? | 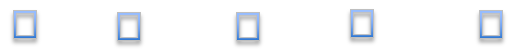 |
| Is your child willing to wear the brace? | 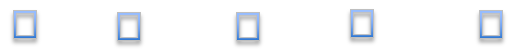 |
| Does your child complain about the brace? | 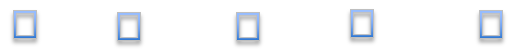 |
| If your child is unwilling, what do you think could be the reason (briefly specify)? |  |
