# Supplementary material for: Understanding bracing outcomes in adolescents with idiopathic scoliosis: a mixed-methods approach
Source: Front Rehabil Sci. 2025 Jul 23;6:1625736. doi: 10.3389/fresc.2025.1625736 (PMC12325295; doi:10.3389/fresc.2025.1625736)
Supplement: Supplementary file 3 [file Datasheet3.docx]

**SEMI-STRUCTURED INTERVIEW GUIDE**

**Title:** Experiences and Perceptions of Brace Use Among Individuals with AIS

**Introduction Script:**

Thank you for agreeing to participate in this interview. I’m interested in learning about your experiences with wearing a scoliosis brace. Please feel free to share anything you think is important. There are no right or wrong answers, and your responses will remain confidential.

**Theme 1: Daily Life Activities**

1. Can you describe how wearing the brace has affected your daily routine (e.g., school, home life)?

2. Have you experienced any difficulties with eating, dressing, or using the bathroom while wearing the brace?

3. In what ways has the brace impacted your clothing choices or body image?

4. How has wearing a brace influenced your interactions with friends or family?

5. Have you felt self-conscious or uncomfortable in public because of the brace?

6. How do you feel emotionally when wearing your brace?

**Theme 2: Materials of the Brace**

7. How would you describe the material and physical comfort of the brace?

8. Have you experienced sweating, pain, or skin irritation due to the brace?

9. Has the brace affected your sleep quality?

10. What do you think about the design, appearance, or material of the brace?

**Theme 3: Use of the Brace**

11. Can you describe your routine for wearing the brace? Are there times when you choose not to wear it?

12. Did events like the pandemic affect how regularly you used the brace?

13. Have you experienced any issues with the brace’s straps or fitting?

14. If you could change anything about your brace, what would it be?

15. What advice would you give to someone who has just started wearing a brace?

**Closing Statement:**

Thank you for sharing your experiences. Your insights will help us better understand what it’s like to live with a brace and may help improve brace treatment for others in the future.
